# Differences between hemispheres and in saccade latency regarding volleyball athletes and non-athletes during saccadic eye movements: an analysis using EEG

**DOI:** 10.1055/s-0043-1772830

**Published:** 2023-10-18

**Authors:** Renan Vicente, Juliana Bittencourt, Élida Costa, Eduardo Nicoliche, Mariana Gongora, Jessé Di Giacomo, Victor Hugo Bastos, Silmar Teixeira, Marco Orsini, Henning Budde, Mauricio Cagy, Bruna Velasques, Pedro Ribeiro

**Affiliations:** 1 Universidade Federal do Rio de Janeiro, Escola de Educação Física e Desportos, Rio de Janeiro RJ, Brazil.; 2 Universidade Federal do Rio de Janeiro, Instituto de Psiquiatria, Rio de Janeiro RJ, Brazil.; 3 Universidade Veiga de Almeida, Rio de Janeiro RJ, Brazil.; 4 Instituto Federal de Educação, Ciência e Tecnologia do Rio de Janeiro, Rio de Janeiro RJ, Brazil.; 5 Universidade Federal do Piauí, Departamento de Fisioterapia, Teresina PI, Brazil.; 6 Universidade Federal Fluminense, Hospital Universitário Antônio Pedro, Niterói RJ, Brazil.; 7 Medical School Hamburg, Faculty of Human Sciences, Hamburg, Germany.; 8 Reykjavik University, Department of Sport Science, Reykjavik, Iceland.; 9 Universidade Federal do Rio de Janeiro, Departamento de Engenharia Biomédica, Rio de Janeiro RJ, Brazil.

**Keywords:** Saccades, Athletes, Electroencephalography, Reaction Time, Movimentos Sacádicos, Atletas, Eletroencefalografia, Tempo de Reação

## Abstract

**Background** The saccadic eye movement is responsible for providing focus to a visual object of interest to the retina. In sports like volleyball, identifying relevant targets quickly is essential to a masterful performance. The training improves cortical regions underlying saccadic action, enabling more automated processing in athletes.

**Objective** We investigated changes in the latency during the saccadic eye movement and the absolute theta power on the frontal and prefrontal cortices during the execution of the saccadic eye movement task in volleyball athletes and non-athletes. We hypothesized that the saccade latency and theta power would be lower due to training and perceptual-cognitive enhancement in volleyball players.

**Methods** We recruited 30 healthy volunteers: 15 volleyball athletes (11 men and 4 women; mean age: 15.08 ± 1.06 years) and 15 non-athletes (5 men and 10 women; mean age: 18.00 ± 1.46 years). All tasks were performed simultaneously with electroencephalography signal recording.

**Results** The latency of the saccadic eye movement presented a significant difference between the groups; a shorter time was observed among the athletes, associated with the players' superiority in terms of attention level. During the experiment, the athletes observed a decrease in absolute theta power compared to non-athletes on the electrodes of each frontal and prefrontal area.

**Conclusion** In the present study, we observed the behavior of reaction time and absolute theta power in athletes and non-athletes during a saccadic movement task. Our findings corroborate the premise of cognitive improvement, mainly due to the reduction of saccadic latency and lower beta power, validating the neural efficiency hypothesis.

## Resumo

**Antecedentes** O movimento ocular sacádico é responsável por dar foco a um objeto visual de interesse para a retina. Em esportes como o vôlei, identificar alvos relevantes o mais rápido possível é essencial para se ter um desempenho magistral. O treinamento melhora as regiões corticais subjacentes à ação sacádica, e permite um processamento mais automatizado em atletas.

**Objetivo** Investigamos as mudanças na latência durante o movimento ocular sacádico e a potência teta absoluta nos córtices frontal e pré-frontal durante a execução da tarefa de movimento ocular sacádico em atletas e não atletas de voleibol. Nossa hipótese é a de que a latência sacádica e a potência teta seriam menores em atletas devido ao treinamento e ao aprimoramento perceptivo-cognitivo em jogadores de voleibol.

**Métodos** Ao todo, 30 voluntários saudáveis foram recrutados para este estudo: 15 atletas de voleibol (11 homens e 4 mulheres; idade média: 15,08 ± 1,06 anos) e 15 não atletas (5 homens e 10 mulheres; idade média: 18,00 ± 1,46 anos). Todas as tarefas foram realizadas simultaneamente com o registro do sinal eletroencefalográfico.

**Resultados** O resultado da latência do movimento ocular sacádico apresentou diferença significativa entre os grupos, sendo observado menor tempo entre os atletas, associado à superioridade dos jogadores em termos de nível de atenção. Durante o experimento, nos eletrodos de cada área frontal e pré-frontal, observou-se uma diminuição na potência teta absoluta nos atletas em comparação aos não atletas.

**Conclusão** Neste estudo, observou-se o comportamento do tempo de reação e da potência teta absoluta em atletas e não atletas durante uma tarefa de movimento sacádico. Nossos achados corroboram a premissa de melhora cognitiva, principalmente pela redução da latência sacádica e menor potência beta, o que valida a hipótese de eficiência neural.

## INTRODUCTION

The saccadic eye movements are fast and precise oculomotor shifts responsible for locating external visual targets, bringing the objects of interest to the retina. 1 2 In a visual search task, the focus of attention is usually determined by the fixation point, and, in this scenario, one observes the need to effectively concentrate on the performance of many sports in which information is relevant. 3 4 Perceptual ability is linked to the athlete's ability to select critical components within a dynamic environment. 5 6 Experienced athletes respond efficiently to information that is relevant to their task, while leaving irrelevant and distracting information unattended. 7 8 Thus, specialized neural processing favors their superior abilities. 4 The ball in movement and the position of the positioning are starting situations in which the athletes must use saccadic eye movements to have an efficient performance; therefore, a relationship is observed between the ease of fixation of the saccade and the sports performance. 9

During the planning and execution of saccadic eye movements, the prefrontal and frontal cortices are involved, causing changes in cortical electrical activity. These changes can be observed through quantitative electroencephalography (EEG). 10 11 12 Investigations point to the theta frequency band (range: 4 Hz to 7 Hz) as the neural correlate of the integration of sensory information to a motor response in the production of voluntary movement. 13 14 Theta is widely distributed through the brain, reflecting actions in the cortex, specifically during complex executive processes such as attention mechanisms, spatial navigation, coding, and memory retrieval. 15 16

Velasques et al. 17 investigated the absolute theta power on the frontal cortex during the planning, execution, and cognitive control of saccadic eye movements. They have shown that the presentation of the stimulus induces distinct patterns of cortical activation between the left and right hemispheres. Moreover, Jafarzadehpur et al. 18 examined the visual accommodation capacity of non-players, beginner volleyball players, and intermediate and advanced volleyball players. Significant differences between non-players and beginners were detected compared to the intermediate and advanced group. In addition, differences in saccadic eye movement have been observed between non-athletes, badminton players, and squash players. However, although the role of theta activity in the planning and execution of saccadic eye movement tasks and distinctions in habituation between athletes and non-athletes have been demonstrated, no experiment has directly investigated the electrophysiological differences between athletes and non-athletes during a saccadic eye movement task.

Therefore, the present experiment aims to directly address this issue by analyzing saccade latency and the absolute theta power in athletes and non-athletes. We want to specifically compare the differences in the average latency during the saccadic eye movement and interhemispheric changes in EEG activity between athletes and non-athletes. We hypothesize a decrease in saccade latency and the theta frequency band in athletes in the frontal and prefrontal regions because of the volleyball training and the perceptual-cognitive expertise of the players.

## METHODS

### Sample

We recruited 30 volunteers: 15 volleyball players (11 men and 4 women; mean age: 15.08 ± 1.06 years) and 15 non-athletes for the control group (5 men and 10 women; mean age: 18.00 ± 1.46 years). The athletes had about 4 years of playing experience, with an average of 15 hours a week of training at elite national-level teams within their age group, while the non-athletes participated in physical activities in high school. All participants had normal vision and no sensory, cognitive, motor, or attentional deficits that would affect the saccadic eye movement. All individuals were right-handed according to the Edinburgh Inventory. 19 In addition, they had had at least 6 to 8 hours of sleep the day before the experiment was performed. The participants signed a consent form that described in detail the experimental procedure. Those responsible for individuals under the age of 18 years signed the consent form. The study was approved by the Ethics Committee of Instituto de Psiquiatria da Universidade Federal do Rio de Janeiro (IPUB-UFRJ) (CAAE: 94619218.3.000.5257), according to the principles of the Declaration of Helsinki (FR 233406) .

### Experimental procedure

The room used to capture the electroencephalographic signal was sound protected, and during data acquisition, the room's brightness was reduced to minimize sensory interference. The individuals sat comfortably in a chair with arm support to minimize muscle artifacts. A bar made up of 13 light-emitting diodes (LEDs) was placed in front of the participants and positioned at the level of their eyes. The bar was composed of six LEDs on the left side of fixation and another six LEDs on the right side, and a bicolored central warning LED. The subjects were within 100 cm of the LED bar. A computer program controlled the bar and determined the presence of the stimulus. The participants were asked to keep their eyes fixed on the center of the bar and to slide them when they noticed that one of the diodes was illuminated; then, they were instructed to follow the LEDs with their eyes so that their heads remained static. The paradigm constituted the realization of a fixed condition of presentation of the luminous stimulus. In this situation, the target stimulus alternated from a predetermined two-point position, that is, the six LEDs on each side of the bar (left or right). This condition was characterized by the predictability of the appearance of the stimulus in time and space, being considered directed by memory. The LEDs blinked alternately between the left and right sides of the bar. The flash of each LED lasted for 250 ms, and the interval between flashes was of 2 s. Each participant was submitted to six blocks of stimulation and two resting periods (without stimulus), one before starting the task and another one after the end. Each block consisted of 20 tracks, with 10 LEDs on the right side of the bar and 10 on the left side, from the warning in the center of the bar.

### Acquisition of electroencephalographic data

The Braintech 3000 device (EMSA Equipamentos Médicos Ltda., Rio de Janeiro, RJ, Brazil) was used to capture the electroencephalographic signal, using a 20-channel analog-digital converter (A/D) board. A capture program, called EEG_Captação (created using the Delphi 5.0 software [Embarcadero Technologies, Austin, TX, United States]), produced at the Brain Mapping and Sensory-Motor Integration Laboratory at UFRJ, was used to acquire the electroencephalographic signal and control the LED lighting. The EEG signal was amplified with a gain of 22 thousand, analogously filtered between 0.1 Hz (high pass) and 100 Hz (low pass), and the sampling was 200 Hz. A 60-Hz digital notch filter was used. The International Federation's 10-20 electrode system (Jasper, 1958) was used to place 20 electrodes along the scalp (areas: frontal, central, temporal, parietal, and occipital) and an electrode in each ear (lobe). The electrodes were mounted on a nylon cap, produced at the same laboratory as mentioned before, with prefixation of the system 10-20. The earlobes were used as a reference (binaural). The impedance of each electrode was maintained between 5 kΩ and10 kΩ. The acquired data had a total amplitude (peak to peak) < 70μV.

### Acquisition of the saccadic eye movement

The ocular electrical activity, or electrooculogram (EOG), was estimated by placing 2 electrodes with 9 mm in diameter mounted bipolarly; they were placed in the outer corner of the left and right eyes that recorded the horizontal eye movements (hEOG).

### Data processing and analysis

In order to remove the possible artifacts produced by the task (blinking, muscular activity, and artifacts related to saccade), we applied the independent component analysis (ICA). The data, collected using the biauricular reference, were transformed (re-referenced) using the average reference; then, they were conducted to eliminate artifacts through ICA. Removed by visual inspection, the tracks that demonstrated the blink of an eye and artifacts related to the saccade, as well as the components that showed blinking and artifacts related to “contamination” of the saccade through ICA, were excluded. The number of samples was of 800 (4 × 200 Hz), with a rectangular window. The quantitative EEG parameters were extracted with a time window of 500 ms before the presentation of the stimulus, and 500 ms after the trigger (moment 1 and moment 2 respectively). After that, all EEG trials were visually controlled, and if there was still a “contaminated” track with muscle artifacts, it was discarded. To test the stationarity of the signal, the Runs test and Reverse Arrangement test was applied. Significantly, the null hypothesis of the stationary process was accepted for every 4 s (period duration in this period).

### Statistical analysis

The statistical analysis was performed using the IBM SPSS Statistics for Windows (IBM Corp., Armonk, NY, United States) software, version 21.0, and saccade latency and absolute theta power (4 Hz to 7 Hz) were the 2 dependent variables of interest. We used a semiautomated method to detect saccade latency; in particular, the saccades were determined at the start of the curve's inflection point, consistently recognized from visual inspection. The saccade was separated 500 ms after the presentation of the stimulus. The highest velocity (first derivate) was detected at the deflection point. From the moment the LEDs are lit, we define a period of 500 ms to seek the EOG inflection and mark the point with the highest ‘‘acceleration’' or the initiation (second derivate) of the EOG signal. Saccades with latencies shorter than 100 ms and longer than 400 ms were not considered.

The independent samples *t* -test (non-athletes and volleyball athletes) was applied to analyze the average change in latency of the saccadic eye movement. In addition, the electrophysiological analysis of the absolute theta power was performed through two-way analysis of variance (ANOVA) determined by two factors: group (non-athletes and volleyball athletes) and momentum (before and after the stimulus to the fixed condition during the task of saccadic eye movement). For those electrodes in which we found an interaction between factors, a paired *t* -test was applied to examine the possible differences. We estimated the effect size as partial squared eta (ƞ ^2^ p), and we calculated the statistical power and the 95% confidence interval (95%CI) for the dependent variables. Each electrode was seen separated precisely to avoid a type I error. Significance was set at *p* ≤ 0.05 for all analyses.

## RESULTS

### Behavioral measures

**Figure 1:**
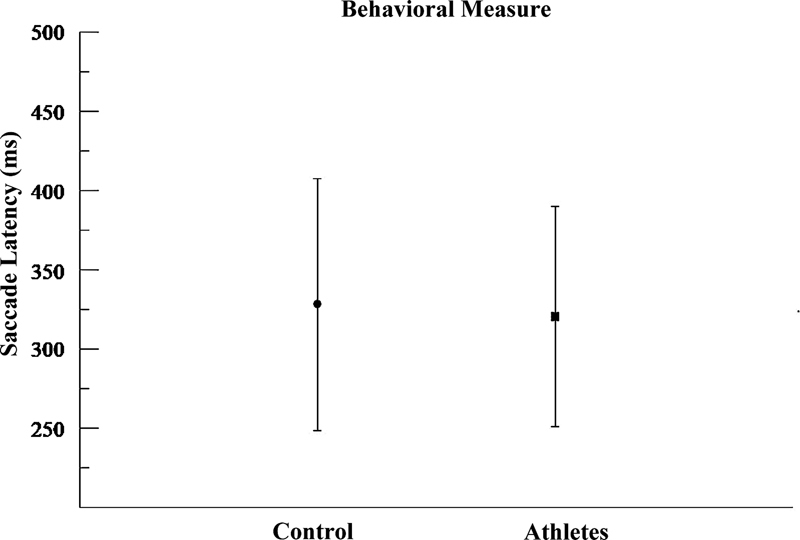
Mean and standard deviation of values for saccade latency (ms) for the two groups (saccade latencies are shown separately for each group). The statistical analysis revealed differences between the group athletes and the control group ( *p* = 0.042).

The independent samples *t* -test for the “group” factor (non-athletes and volleyball athletes) was performed to examine changes in saccadic eye movement. We observed that the latency in the “fixed” paradigm of the task of saccadic eye movement presented a significant difference (t [1.1488] = 2.039; *p* = 0.042). We identified a decrease in the average time for the group of athletes (mean = 320.369 ± 69.382 ms) compared to the group control of non-athletes (mean = 328.250 ± 79.475 ms). The mean difference in reaction time between the control group and athletes is shown in Figure 1 .

### Electrophysiological parameters

**Figure 2:**
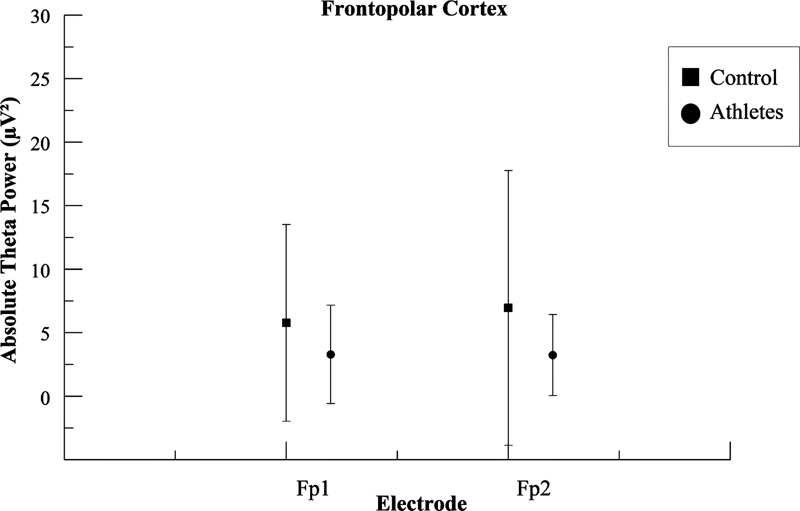
Mean and standard deviation values for absolute theta power (μV ^2^ ) in the frontopolar cortex. The figure illustrates the difference among groups for each pair of electrodes located on the frontopolar cortex. For Fp1, the statistical analysis revealed differences in the left frontopolar cortex between the athlete group and the control group ( *p* < 0.001); for Fp2, the statistical analysis revealed differences in the right frontopolar cortex between the athlete group and the control group ( *p* < 0.001).

**Figure 3:**
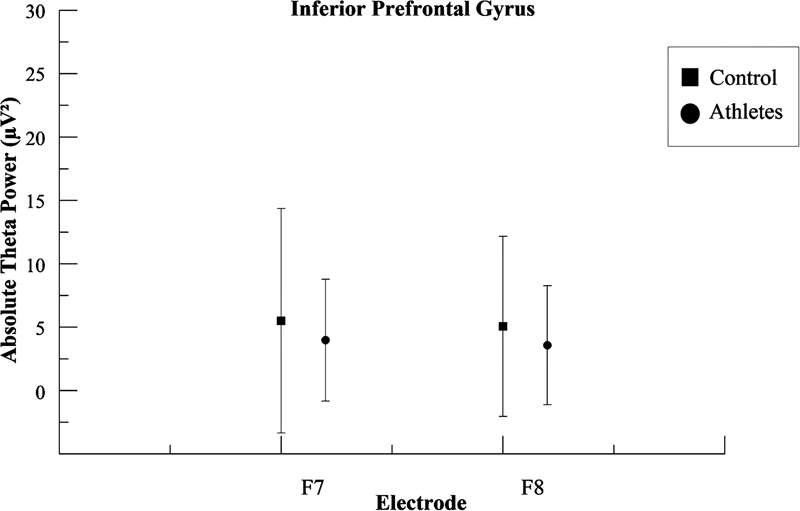
Mean and standard deviation values for the absolute theta power (μV ^2^ ) in the inferior prefrontal gyrus. The figure illustrates the difference among groups for each pair of electrodes located on the inferior prefrontal gyrus. For F7, the statistical analysis revealed differences in the left inferior prefrontal gyrus between the athlete group and the control group ( *p* < 0.001); for F8, the statistical analysis revealed differences in the right inferior prefrontal gyrus between the athlete group and the control group ( *p* < 0.001).

**Figure 4:**
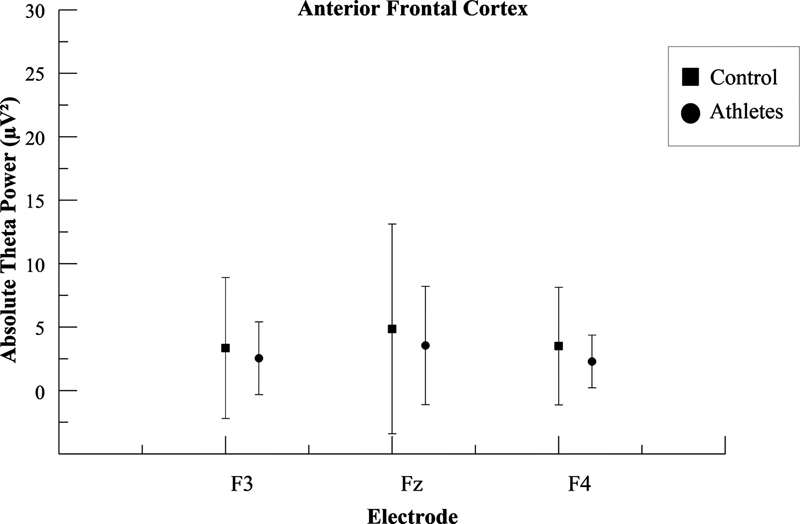
Mean and standard deviation values for the absolute theta power (μV ^2^ ) are shown separately for the three frontal electrodes (F3, Fz, and F4) on the anterior frontal cortex. For F3, the statistical analysis revealed differences in the left anterior frontal cortex between the athlete group and the control group ( *p* < 0.001); for Fz, the statistical analysis revealed differences in the midline anterior frontal cortex between the athlete group and the control group ( *p* < 0.001); for F4, the statistical analysis revealed differences in the right anterior frontal cortex between the athlete group and the control group ( *p* < 0.001).

The two-way ANOVA ( *p* ≤ 0.05) indicated a significant effect on absolute theta power for the “group” factor. We observed a decrease in the theta frequency in the group of athletes compared to the non-athletes in the electrodes: Fp1 (F [1.3367] = 138.815; *p* < 0.001; ƞ ^2^ p = 0.040; power = 99.9%); Fp2 (F [1.3376] = 182.930; *p* < 0.001; ƞ ^2^ p = 0.051; power = 99.9%); F7 (F [1.3393] = 39.067; *p* < 0.001; ƞ ^2^ p = 0.011; power = 99.6%); F8 (F [1.3380] = 51.138; *p* < 0.001; ƞ ^2^ p = 0.015; power = 99.9%); F3 (F [1.3389] = 27.661; *p* < 0.001; ƞ ^2^ p = 0.008; power = 90.6%); Fz (F [1.3382] = 31.768; *p* < 0.001; ƞ ^2^ p = 0.009; power = 97.3%); and F4 (F [1.3386] = 96.518; *p* < 0.001; ƞ ^2^ p = 0.028; power = 99.9%). Figures 2 , 3 and 4 show the mean and standard deviation (SD) differences in absolute theta power for the two groups in the frontopolar cortex the inferior prefrontal gyrus, and the anterior frontal cortex respectively.

## DISCUSSION

The experiment proposed to analyze saccade latency (behavioral measurement) and the absolute theta power (electrophysiological measurement) in the prefrontal and frontal cortices during the execution of the “fixed” paradigm in the task of saccadic eye movement.

### Behavioral measure

The behavioral analysis revealed a significant difference expressed by a shorter average saccade latency for the athletes. A decrease in latency among the athletes is mainly associated with their superiority in terms of cognitive abilities. Such a finding supports our hypothesis because of the volleyball players' excellent proficiency in visual information fixation and, consequently, more extraordinary visual allocation ability. 5 18 20 These play a crucial role in performing sports tasks, including information processing speed and working memory capacity. 21 22

The neural circuits for the initiation of the saccadic eye movement and the development of attention are interconnected. 23 Our results indicate that the athletes responded to the task of saccadic eye movement more efficiently, mainly associated with decreased attentional processes. 24 25 In such a way, continuous training results in shortening of the preparation of saccadic eye movements, and, as a result, athletes move their eyes towards a visual target more quickly, reducing saccade latency.

### Electrophysiological parameters

#### Anterior prefrontal cortex

The anterior prefrontal cortex is located anatomically in the anterior frontal region of the cerebral cortex (areas 9 and 10 of Brodmann). Responsible for planning, organization, and motor representation, it is essential in integrating sensory and mnemonic information. 26 27 The pair of electrodes (Fp1 and Fp2) presented similar results for the main group effect. A decrease in the absolute theta power in the left electrode (Fp1) was observed for the group of athletes. Despite the current understanding that there is a complementarity between the hemispheres, the left is known for its specialized role in analytical aspects and motor functions, such as the preparation and execution of saccadic eye movements. 28

This decrease in theta can represent a specialization of the athletes in responding to the demands of the experimental task, producing superior performance in the allocation of perceptual processes, in the reaction time, and in the behavior of the saccadic eye movement. 29 Acquiring motor skills leads to plastic changes in the cortical activation of broad neural connections, leading to functional specificity. Another experiment 7 that analyzed the differences in theta in basketball players during the aiming period between successful and unsuccessful free throws investigated this qualified processing of the athletes. The authors 7 found that the group of experienced athletes demonstrated more cortical activity in the frontal region than the novices, suggesting the superiority of this group in concentrating on the relevant aspects of the specific behavior. 7

The right hemisphere (Fp2) is more related to mnemonic processes, such as working memory, 6 which is a skill that individuals acquire to satisfy the demands of cognitive activity in a domain. 30 Theta reduction can be explained by the functional plasticity underlying cognitive-motor training in athletes in the fixed condition of the experiment. This principle can be observed in the reduction of the power of cortical activity in specific brain regions since a skill becomes less controlled and automated after learning a complex task. 31 The result found in the Fp2 electrode supports the hypothesis of neural efficiency. The neural advantages observed in athletes are characterized as neural efficiency, suggesting lower energy expenditure and better performance during task performance since a skill becomes less controlled and automated after learning a complex task. 31 Volleyball players have better cognitive abilities compared to non-athletes. 32 33

#### Inferior prefrontal gyrus

Traditionally, the inferior prefrontal gyrus is associated with language and semantic functions, as well as with working memory 34 and long-term memory processes. 27 The data of the present study suggest that prefrontal gyration is not limited to aspects of speech and understanding of speech. A decrease in the theta power in the athletes was verified for the electrode located in the left hemisphere, characterized by analytical processes (F7). The decrease in F7 can be explained by the athletes' ability to detect, retain, and allocate attention to information during the experimental protocol. Repeating the motor gesture during training leads to plastic adaptations in the cognitive-motor system, improving executive functions, such as working memory and attention. Successful players have significant neuroplastic advantages in neural processing compared to novices or non-practitioners in cognitive-motor tasks. The athletes skillfully perceive and determine the ball's position and other players by focusing on relevant stimuli. 35

The right inferior prefrontal gyrus (Brodmann area 45) has functions related to episodic and prospective memory. Episodic memory is the ability to recall specific events, and prospective memory is the ability to evaluate and plan future actions. 36 Our results show a decrease in the absolute theta power in the group of athletes in electrode F8. The results also suggest a greater involvement of episodic and prospective memory over the fixed paradigm in the task of saccadic eye movement associated with functional plasticity, indicating the improvement of the executive functions in the group of athletes according to the neural efficiency hypothesis.

#### Anterior frontal cortex

Located in the premotor cortex, the anterior frontal cortex is also known as the frontal visual field. This region plays an essential role in the control of saccadic eye movements. 17 We observed significant results in the electrodes of the anterior frontal cortex (F3, Fz, and F4). A possible interpretation for such a finding can be attributed to the absence of correction and modulation of motor programs due to the fixed task of experiment. 5 Furthermore, the frontal theta midline frequency (Fz) is a sustained attention indicator candidate. 7 Our data showed a decrease in the absolute theta power in the group of athletes compared to non-athletes, suggesting that expertise practically modified neural structures underlying sustained attention. Therefore, the results of the present study indicate a cognitive enhancement of volleyball players in applying sustained attention at the beginning of the saccadic eye movement.

### Limitations of the present study

Although the present study has shown interesting results, some limitations are listed below:

- We initially limited the electroencephalographic analysis to only 20 channels, thus leaving adequate spatial sampling for future analyses;
- Claims regarding EEG activity between athletes and non-athletes should be more moderate; further analysis with another modality of elite athletes is needed;
- Our experimental task was characterized by the predictability of the location and direction of the saccadic stimulus (guided by memory). Therefore, future works should investigate another paradigm (such as visually guided) in order to better elucidate the cortical dynamics between athletes and non-athletes;
- Based on the literature, which indicates that the practice of physical exercise is a neuroenhancer, in future studies, it is also essential to consider the use of event-related potentials (ERPs) associated with cognition before and after physical training.

In conclusion, the present study examined differences in latency and absolute theta power between athletes and non-athletes during a saccadic eye movement task. In general, our results support the premise of cognitive improvement of the players in the experiment. The results supported the principle of neural efficiency associated with plasticity in the group of players on the complex executive functions. The neural correlates of prefrontal and frontal regions are critical in identifying more talented players, or they could be used for pre- to posttraining performance feedback.
